# Gut microbiota induces hepatic steatosis by modulating the T cells balance in high fructose diet mice

**DOI:** 10.1038/s41598-023-33806-8

**Published:** 2023-04-24

**Authors:** Xiaoqiong Zhou, Xianjuan Zhang, Delei Niu, Shuyun Zhang, Hui Wang, Xueming Zhang, Fulong Nan, Shasha Jiang, Bin Wang

**Affiliations:** 1 Department of Pathogenic Biology, College of Basic Medicine, Qingdao University, Qingdao, China; 2 Department of Special Medicine, College of Basic Medicine, Qingdao University, Qingdao, China

**Keywords:** Gastroenterology, Medical research

## Abstract

Metabolic diseases are often associated with high fructose (HF) consumption. HF has also been found to alter the gut microbiota, which then favors the development of nonalcoholic fatty liver disease. However, the mechanisms underlying of the gut microbiota on this metabolic disturbance are yet to be determined. Thus, in this study, we further explored the effect the gut microbiota concerning the T cells balance in an HF diet mouse model. We fed mice 60% fructose-enriched diet for 12 weeks. At 4 weeks, HF diet did not affect the liver, but it caused injury to the intestine and adipose tissues. After 12 weeks, the lipid droplet aggregation was markedly increased in the liver of HF-fed mice. Further analysis of the gut microbial composition showed that HF decreased the *Bacteroidetes*/*Firmicutes* ratio and increased the levels of *Blautia*, *Lachnoclostridium*, and *Oscillibacter*. In addition, HF can increase the expression of pro-inflammatory cytokines (TNF-α, IL-6, and IL-1β) in the serum. T helper type 1 cells were significantly increased, and regulatory T(Treg) cells were markedly decreased in the mesenteric lymph nodes of the HF-fed mice. Furthermore, fecal microbiota transplantation alleviates systemic metabolic disorder by maintaining liver and intestinal immune homeostasis. Overall, our data indicated that intestinal structure injury and intestinal inflammation might be early, and liver inflammation and hepatic steatosis may be a subsequent effect following HF diets. Gut microbiota disorders impairing the intestinal barrier function and triggering immune homeostasis imbalance may be an importantly responsible for long-term HF diets induced hepatic steatosis.

## Introduction

In the last 200 years, fructose consumption diet has increased 100-fold among humans, probably exceeding the capacity of human evolution to make physiologically healthy adaptations. High fructose (HF) consumption is blameworthy for the development and progression of metabolic diseases. In fact, epidemiological studies have shown that HF intake is strongly associated with chronic diseases, including obesity, nonalcoholic fatty liver disease (NAFLD), type 2 diabetes mellitus (T2DM), renal dysfunction, and cardiovascular diseases^1,2^. However, the biological mechanisms underlying the development and progression of these metabolic diseases remain to be controversial.

Gut microbiota has been known to be primarily composed of nine phyla of bacteria and one archaeon, which are capable of synthesizing and secreting essential substances to maintain the normal function of the organs and immune systems, and have a significant impact on the genetic activities and lifestyle of the host^3,4^. The gut microbiome plays an important role in human health. Several pieces of evidence have suggested an intricate linkage between the gut microbiome and the pathogenesis of metabolic diseases^5,6^. Previous studies have indicated that the composition of gut microbiota is related to macronutrient intake^7^. A diet rich in saturated fatty acids can alter the gut microbiota composition and increase the proportion of gram-negative bacteria. This alteration stimulates the production of lipopolysaccharide (LPS),increases intestinal permeability, and leads to metabolic endotoxemia^8^. HF has also been found to alter the gut microbiota, which then favors the development of NAFLD^9^. However, the mechanisms underlying an HF diet affecting NAFLD via the gut microbiome are still unknown. Therefore, in-depth research on the pathogenesis of diet-induced changes in gut microbiota and obesity-related metabolic disorders is of great significance for the prevention and treatment of metabolic diseases in the future.

The crosstalk between gut microbiota and the immune system is intricate and is partially dependent on gut microbial metabolites^10^. Gut microbiota maintains the intestinal mucosal barrier and interacts with the immune system. As the first organ that comes into contact with blood from the intestine, the liver will be deeply influenced by the gut microbiota and its metabolites, and the intestinal leakage and an imbalance of the gut microbiota are the trigger of the pathological reaction of the liver^11^. More studies have examined that high fructose can contribute to hepatic steatosis^12,13^. The innate and adaptive immune systems are both implicated in the pathogenesis of NAFLD, which also involves many organ systems, including a reciprocal relationship between the liver and adipose tissue^14^. Differentially active T-cell subsets such as T helper (Th)1, regulatory T (Treg), and cytotoxic T (Tc) cells play complementary roles in physiologic circumstances, most significantly in the defense against pathogens infections and tumors. These cells also have a role in the development of a number of immune-mediated illnesses. There is growing evidence that Treg cells have a general taming effect on NAFLD^14^. However, the exact role of the intestinal flora concerning these cells still is unclear.

Thus, in this study, we aimed to explore the effect the gut microbiota concerning the T cells balance in an HF diet mouse model. Our results suggested that HF diet caused hepatic steatosis via altering the gut microbiota diversities, destroying the colonic epithelial barrier integrity, and inducing T cells (Th1, Treg, and Tc cells) imbalance. Furthermore, we also indicated that fecal microbiota transplantation (FMT) alleviates systemic metabolic disorder by modulating composition ratio of T cells.

## Results

### Effects of HF diet on body weight and insulin resistance

**Figure 1:**
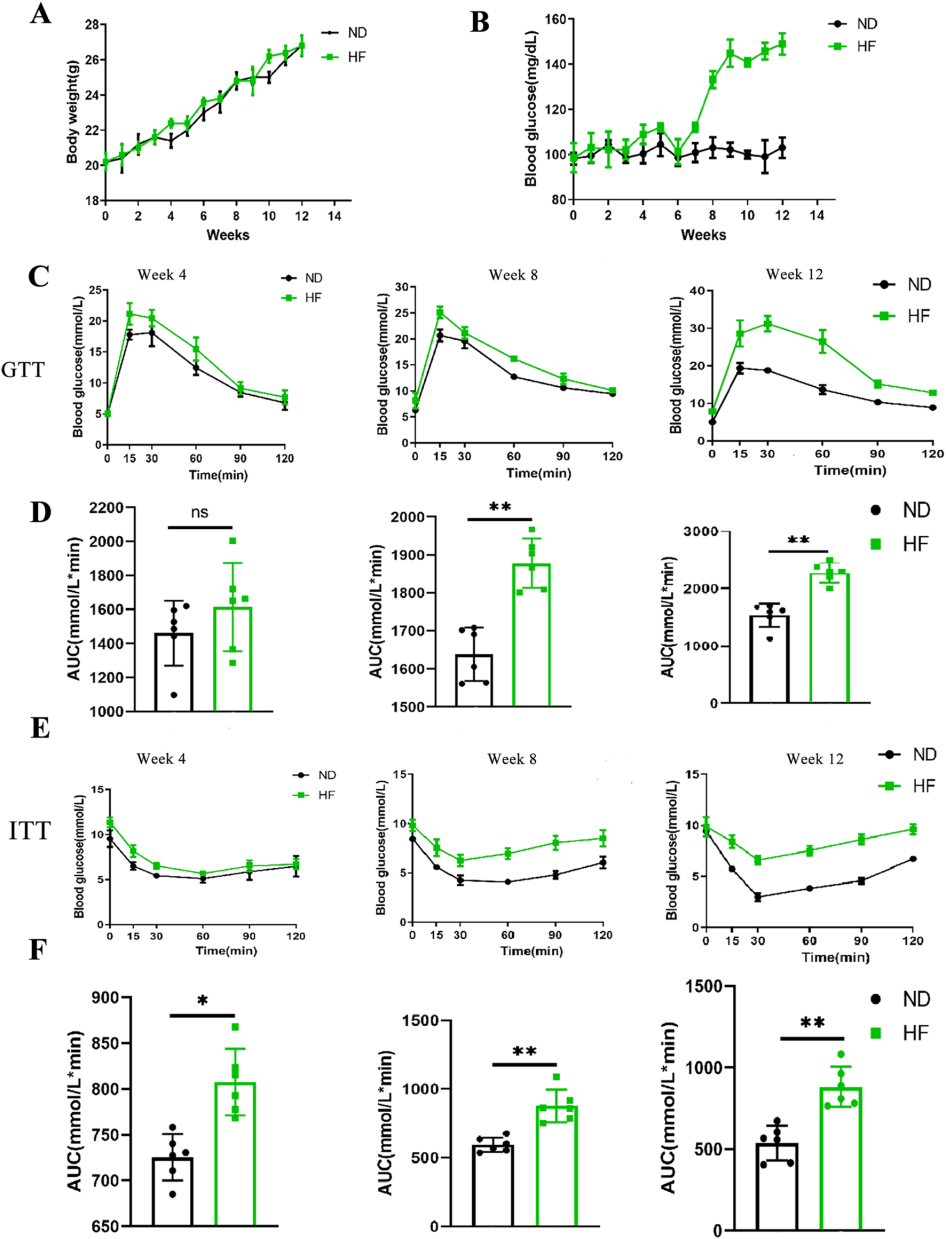
Effects of HF diet on body weight and insulin resistance. (**A**) Weight gain of mice on ND and HF during the 12 weeks of feeding. (**B**) Fasting blood glucose changes during the 12 weeks of feeding. (**C**) Glucose tolerance test, and (**D**) glucose tolerance test calculated AUC measured at 4, 8, and 12 weeks. (**E**) Insulin tolerance test, and (**F**) insulin tolerance test calculated AUC measured at 4, 8, and 12 weeks. The student’s t-test was used to determine statistical significance. *p < 0.05; **p < 0.01.

At the end point of the experiment, the weight of an HF-fed mice was not significantly different from the ND-fed group (Fig. 1A). To investigate the effects of HF on fasting blood glucose (FBG), we have continuously measured the concentration of FBG of mice during the experiment (Fig. 1B). The results showed that there was no significant difference in FBG between the two groups at 4 weeks, while the levels of FBG were increased significantly in the HF-fed mice from 8 to 12 weeks.

To determine whether an HF diet triggers systemic insulin resistance, we continuously monitor changes in glucose tolerance and insulin resistance in mice. The oral glucose tolerance test data revealed that there was no significant difference in glucose tolerance between the two groups of mice at 4 weeks. However, consistent with changes in the FBG levels, an HF diet can markedly increase the glucose intolerance at 8 weeks, which was more pronounced at the 12th week (Fig. 1C,D). The insulin tolerance test data showed that compared to the ND-fed group, the blood glucose levels in mice significantly increased in the HF-fed group after insulin injection (Fig. 1E,F). These data suggest that a long-term HF diet has destroyed the balance of FBG and impaired the insulin resistance without causing obesity in mice, which is aggravated with the prolongation of HF feeding time.

### HF diet Induces hepatic steatosis

**Figure 2:**
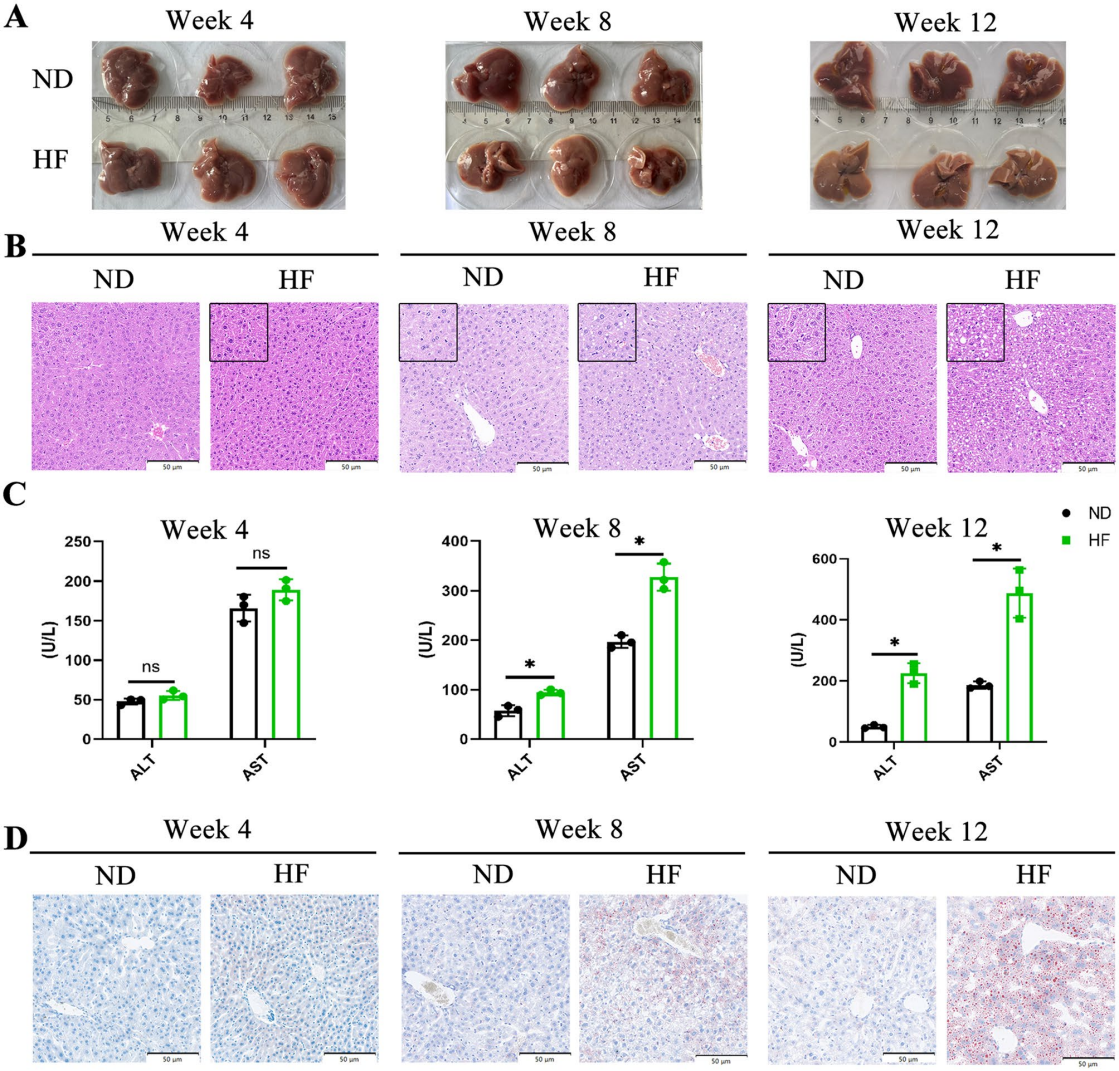
Effects of HF diet on liver morphology, liver histopathological features, serum ALT and AST activities and hepatic lipid accumulation of mice. (**A**) Comparison of liver morphology both two groups of mice at 4, 8, and 12 weeks; (**B**) H&E staining of liver sections. Bar: 50 µm. (**C**) Effect of high fructose on serum ALT and AST activity; (**D**) Oil-Red O staining of liver sections. Bar: 50 µm. The student’s t-test was used to determine statistical significance. *p < 0.05.

To observe the effects of a long-term HF diet on liver, we have collected the liver at stages 4, 8, and 12 weeks. A comparison of the liver morphology showed no significant difference at the 4 weeks, while the livers of the HF-fed mice were friable and soft, and the color changed from dull red to brown from the 8 weeks (Fig. 2A). Liver histological examinations were performed to verify the degree of hepatic steatosis. As shown in Fig. 2B, the H&E staining showed almost no vacuolar degeneration in the liver of the HF-fed mice at 4 weeks. However, the hepatic histology of the HF-fed group mice exhibited significant hepatocyte swelling and degeneration, balloon-like changes, and lipid droplets at 12 weeks. Moreover, an HF diet caused a marked increase in serum AST (aspartate aminotransferase) and ALT (alanine aminotransferase) levels from 8 weeks (Fig. 2C).

Next, we evaluated the hepatic lipid accumulation. As shown in Fig. 2D, the Oil Red O staining indicated that an HF diet could induce lipid accumulations from 8 weeks, and the hepatic steatosis were significantly increased at 12 weeks. Consistently increased plasma concentration of triglyceride (TG) and total cholesterol (TCHO) and the content of TG and TCHO in the liver further supported that an HF diet disturbed host hepatic lipid metabolism (Figures S2A and S2B). Overall, a long-term HF diet induced the development of nonobese fatty liver disease characterized by hepatic steatosis and hepatocellular ballooning.

### Effects of HF diet on white adipose tissue (WAT), brown adipose tissue (BAT), and Pancreas

**Figure 3:**
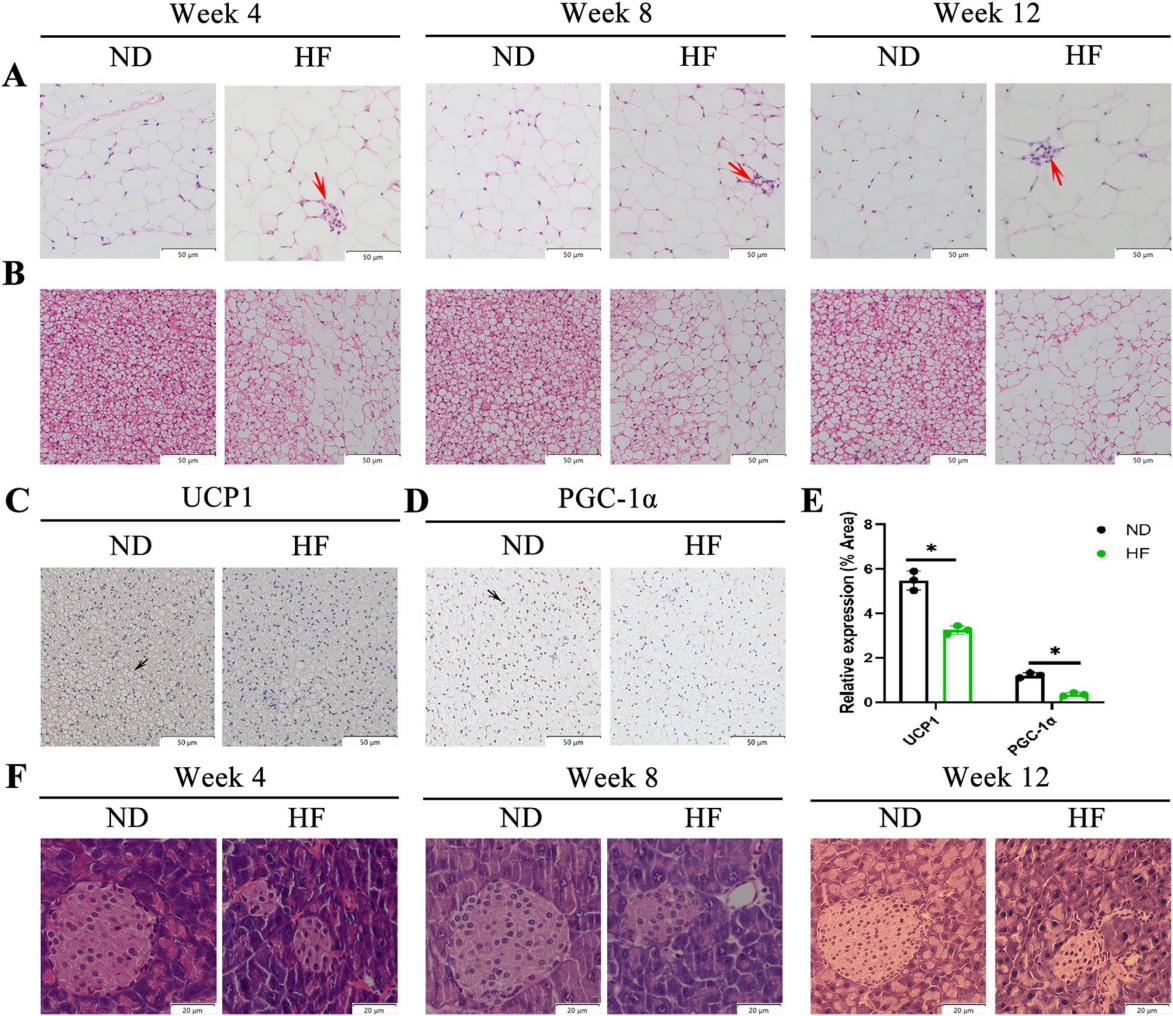
Effects of HF diet on WAT, BAT, and Pancreas. (**A**, **B**) Representative histological results of WAT and BAT by hematoxylin and eosin staining. Inflammatory foci are annotated with red arrows. Bar: 50 µm. (**C**, **D**) BAT sections stained with immunohistochemistry for UCP1 and PGC-1α. Bar: 50 µm. (**E**) Quantification of UCP1 and PGC-1α positive cells. (**F**) Representative H & E images of Pancreas. Bar: 20 µm. The student’s t-test was used to determine statistical significance. *p < 0.05.

As indicated by H&E staining, the histological analysis of WAT and BAT showed that the HF-fed group had significantly increased adipocyte size, markedly reduced cell number per unit area, severe lipid accumulation, and inflammatory foci from 4 weeks (Fig. 3A,B). Moreover, immunohistochemical assessment of the BAT showed the down-regulation of regulatory metabolic factors, including UCP1 and PGC-1α (Fig. 3C–E), the results indicated a decrease in the thermogenic capacity of BAT in the HF diet group. We also performed H&E staining on the pancreas of the mice, as shown in Fig. 3F, the islets were round, and the cells in the islets were evenly arranged in the ND-fed group. However, the islets were oval, and the cross-sectional area of the islets became smaller in the HF-fed group from 4 weeks. These results suggest that WAT, BAT and pancreas were the first to be affected by the HF diet than the liver.

### HF diet decreases the diversity and alters the structure of gut microbiota

**Figure 4:**
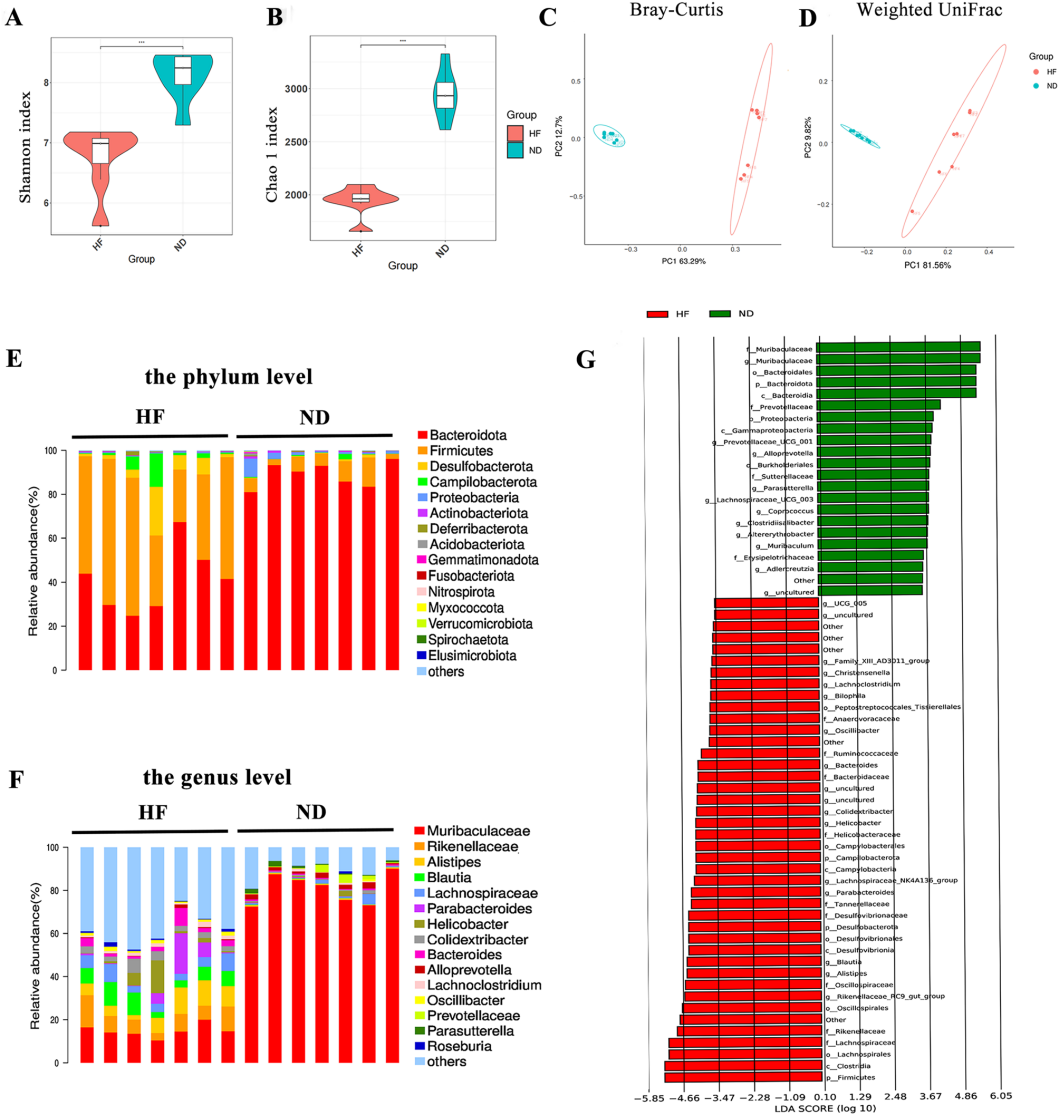
Analysis of the gut microbial community by 16S rRNA pyrosequencing from feces of HF and ND groups after feeding for 4 weeks. α-Diversity was calculated by Shannon index (**A**) and Chao 1 index (**B**). Principal coordinate analysis of (**C**) unweighted UniFrac analysis and (**D**) Bray–Curtis analysis. (**E**, **F**) Relative abundances of a common set of microbial families were compared at the phyla and genus level in HF and ND groups. (**G**) Differences in microbial taxa at all taxonomic levels between HF and ND group were calculated by LDA effect size (LEfSe). Mann–Whitney test was used to determine statistical significance. *p < 0.05; **p < 0.01; ***p < 0.001.

Gut microbiota plays an essential role in the pathogenesis of metabolic diseases. To determine the effects of an HF diet on the gut microbiota composition, we performed 16S ribosomal RNA (rRNA) gene sequencing using fecal samples from a 4-week ND-fed group and the HF-fed group. The richness and alpha diversity analysis exhibited that the Chao1 and Shannon indexes were significantly lower in the HF-fed group than in the ND-fed group (Fig. 4A,B). Bray–Curtis and weighted UniFrac indicators showed that the microbial population β-diversity was significantly altered in the HF-fed group, which was reflected in different clustering patterns on the principal coordinates analysis plot (Fig. 4C,D). Furthermore, the sample microbial community structure revealed analysis showed a significant change in the gut microbiota composition in the HF-fed group compared to the ND-fed group. At the phylum level, the relative abundance of *Firmicutes* increased significantly in the HF-fed group. In contrast, the relative abundance of *Bacteroidetes* decreased significantly in the HF-fed group compared to the ND-fed group (Fig. 4E). The HF-fed group was found to have a higher abundance of *Rikenellaceae*, *Blautia*, *Alistipes*, *Lachnoclostridium*, *Colidextribacter*, *Bacteroides*, and *Oscillibacter*at the genus level than the ND-fed group. In contrast, the ND-fed group had a higher abundance of *Muribaculaceae* and *Alloprevotella* than the HF-fed group (Fig. 4F). We further applied linear discriminant analysis effect size (LEfSe) for differential discriminant analysis to identify actionable microbial taxa that were differentially enriched with high fructose intake (Fig. 4G). *Firmicutes*, *Clostridia*, *Lachnospirales*, and *Rikenellaceae* were among the most abundantly increased bacteria, whereas *Muribaculaceae*, *Bacteroidales*, *Prevotellaceae*, and *Proteobacteria* were among the bacteria most abundantly reduced in HF-fed mice. These results suggest that an HF diet significantly reduced the species richness and diversity of the gut microbiota, leading to an imbalance of fecal microbes.

### HF diet damages the colonic epithelial barrier integrity and causes and intestinal inflammation

**Figure 5:**
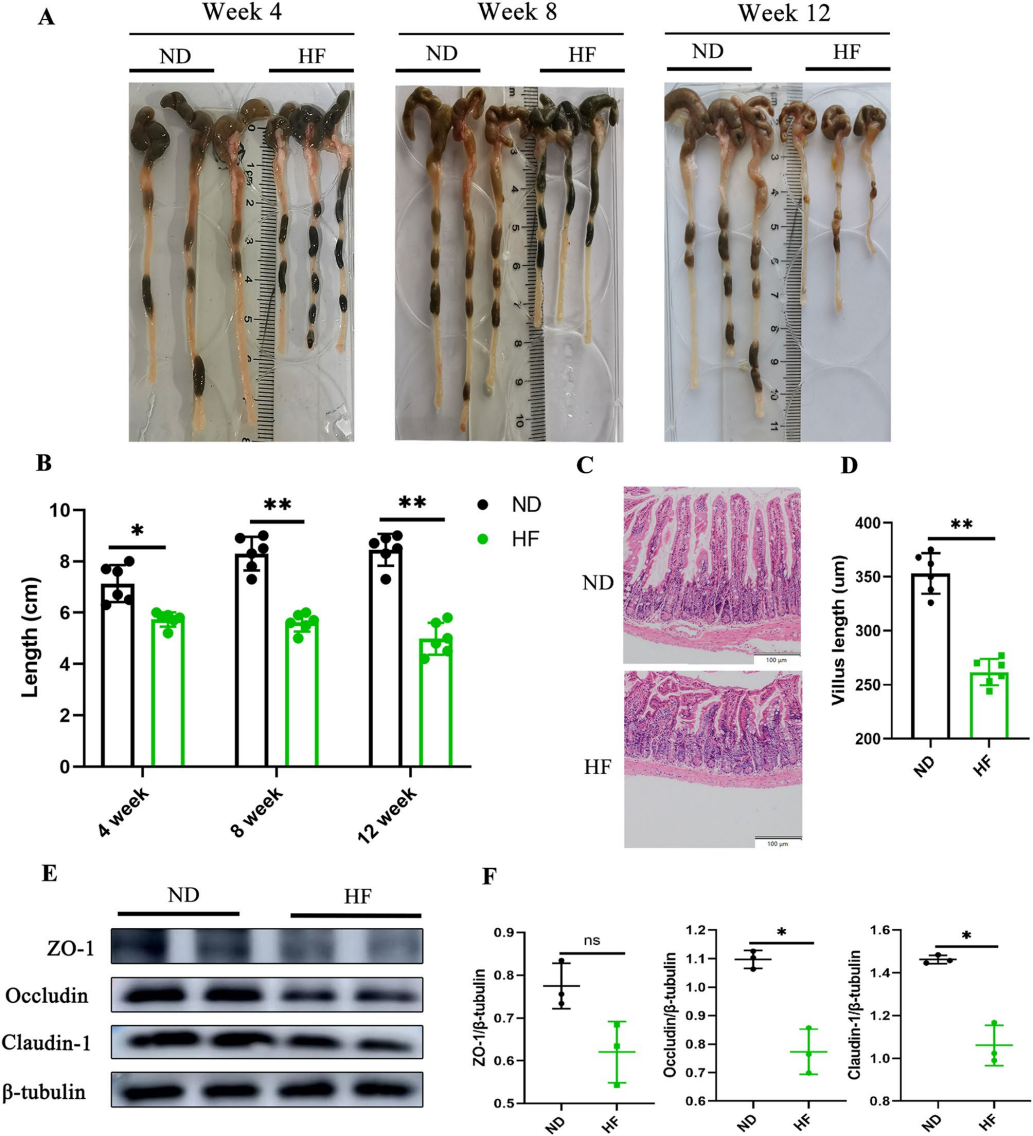
HF diet alters the intestinal morphology and gut permeability. (**A**) Representative images of the colon of mice at 4, 8, and 12 weeks. (**B**) Quantified colon length. (**C**) Representative H&E staining images of villi in the jejunum at 4 weeks. Bar: 100 µm. (**D**) Quantified villus length. (**E**) Representative images of Western blots for tight junction proteins (ZO-1, occludin, and claudin-1) at 4 weeks. (**F**) Gray-scale value analysis of ZO-1, occludin, and claudin-1 at 4 weeks. The student’s t-test was used to determine statistical significance. *p < 0.05; **p < 0.01.

To elucidate the effect of an HF diet on intestinal function, we first have evaluated the overall appearance of the intestine. The results revealed that compared to the ND-fed group, the colon length was obviously shorter in the HF-fed group from 4 weeks (Fig. 5A,B). The H&E staining was used to analyze the length of the intestinal villi. In the small intestine, the results revealed that the length of intestinal villi in the HF-fed group was significantly shorter than the ND-fed group (Fig. 5C,D). Intestinal barrier plays an important role in preventing the spread of potentially harmful pathogens and toxins. Tight junctions (TJs) are known to play an important role in maintaining intestinal barrier integrity, and are composed of a variety of tight junction proteins (transmembrane proteins), such as ZO-1, occludin, and claudin-1. To evaluate the effect of an HF diet on intestinal permeability, the protein expression levels of colonic tight junction proteins were quantified. The results showed that the expression of ZO-1, occludin, and claudin-1 was significantly lower in the HF-fed group than in the ND-fed group (Fig. 5E,F).

**Figure 6:**
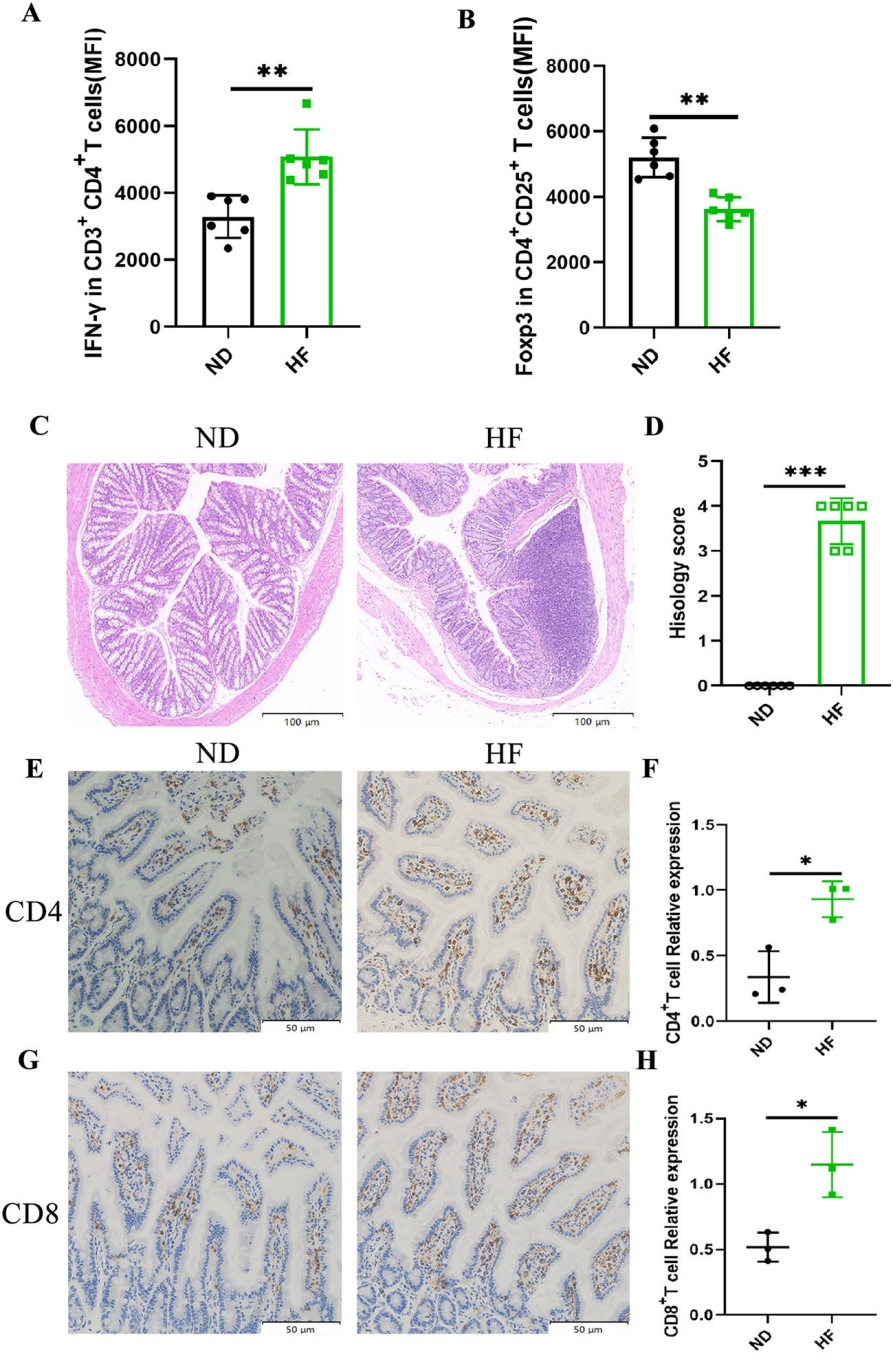
HF diet causes gut inflammation. We gated on mesenteric lymph nodes lymphocytes at 4 weeks and measured the MFI expression level of IFN-γ (**A**) in CD3^+^CD4^+^T cells and Foxp3 (**B**) in Treg cells (CD25^+^Foxp3^+^) by flow cytometry. Colon tissues were examined histologically after (**C**) H&E staining and (**D**) scored for inflammation and epithelial injury. Bar: 100 µm. Immunohistochemistry for 4-week jejunums, with primary antibodies against (**E**) CD4 and (**G**) CD8. Bar: 50 µm. Quantification of (**F**) CD4^+^ and (**H**) CD8^+^ T cells. The student’s t-test was used to determine statistical significance. *p < 0.05; **p < 0.01, ***p < 0.001.

To assess the effect of an HF diet on intestinal inflammation, we have isolated lymphocytes from the mesenteric lymph nodes (MLN) of mice. Flow cytometry analyzed the proportions of pro-inflammatory Th1 cells (CD4^+^IFN-γ^+^) and anti-inflammatory Treg cells (CD25^+^Foxp3^+^). Compared to the ND group, the Th1 cells were significantly increased, and the Treg cells were markedly decreased in the intestinal lymph nodes of the HF-fed mice (Fig. 6A,B and figures S3A and S3B). The colon histopathology that the colons of HF-fed mice exhibited inflammatory cell infiltration and increased epithelial injury score (Fig. 6C,D). The jejunum is the main part of fructose absorption. Therefore, we performed immunohistochemical staining of CD4^+^T cells and CD8^+^T cells in the jejunum (Fig. 6E–H). The results showed increased infiltration of CD4^+^ and CD8^+^T cells in the jejunum of the HF-fed mice. The above results suggest that an HF diet can increase intestinal permeability and promote the intestinal inflammatory response.

### HF diet induces liver inflammation and lipid metabolism disorders

**Figure 7:**
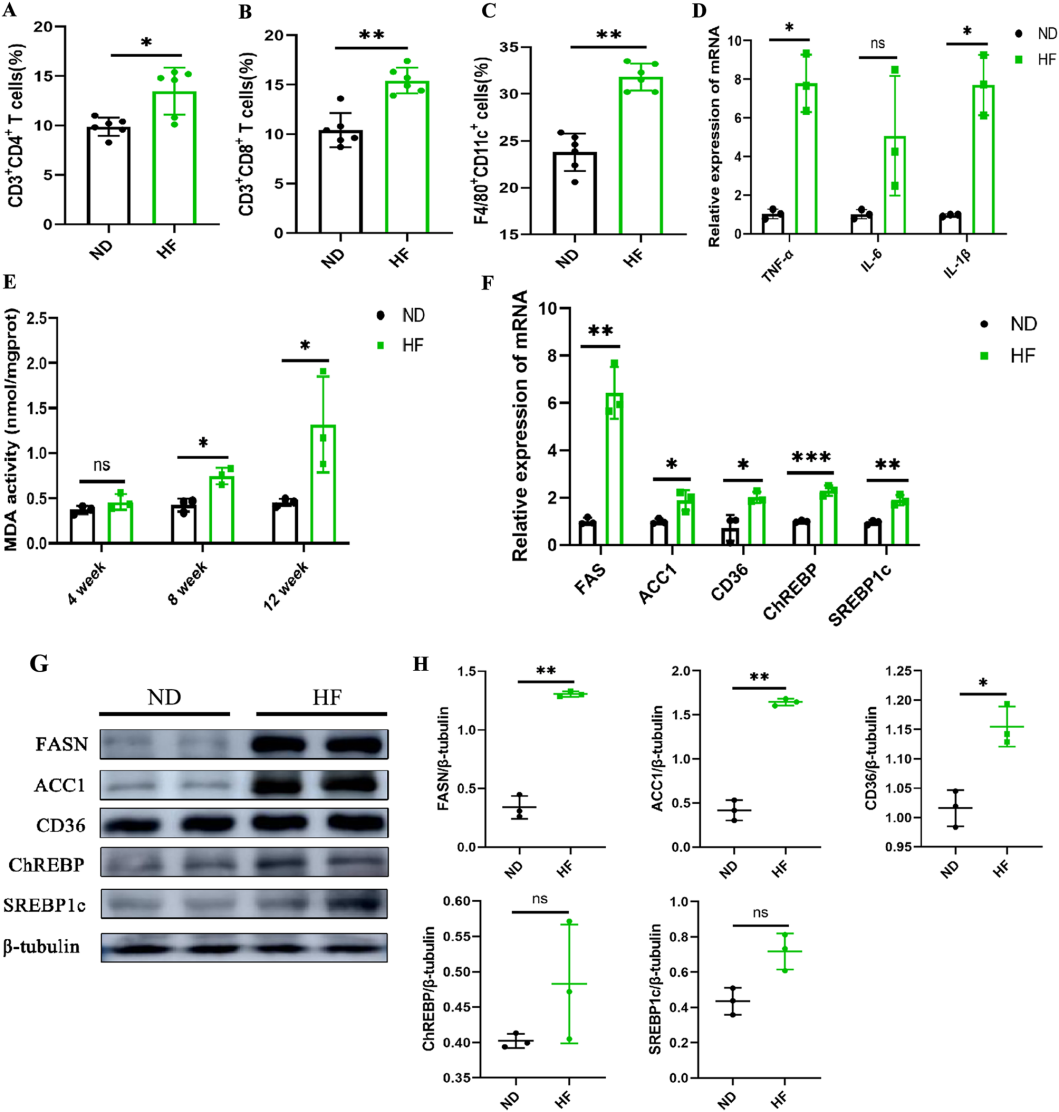
HF Diet Induces Liver Inflammation. The percentage of (**A**) CD3^+^CD4^+^, (**B**) CD3^+^CD8^+^ T cells and (**C**) M1 macrophages (F4/80^+^CD11c^+^) in liver mononuclear cells were detected by flow cytometry at 8 weeks. The cells were analyzed by FACS. (**D**) mRNA expression levels of the TNF-α, IL-6, and IL-1β were measured by RT-qPCR at 8 weeks. (**E**) Liver MDA activity was measured at 4, 8 and 12 weeks. (**F**) mRNA expression levels of the *FAS*, *ACC1*, *CD36*, *ChREBP*, and *SREBP1c* were measured by RT-qPCR. (**G**) Representative images of Western blots for lipid metabolism (FASN, ACC1, CD36, ChREBP, and SREBP1c). (**H**) Gray-scale value analysis of FASN, ACC1, CD36, ChREBP, and SREBP1c. The student’s t-test was used to determine statistical significance. *p < 0.05; **p < 0.01; ***p < 0.001.

Increased intestinal permeability may lead to the entry of endotoxin into the portal vein, which is an essential trigger for the fatty liver formation and liver inflammation^12^. To determine whether ran altered composition of gut microbiota is associated with an HF diet-induced hepatic injury, we isolated mononuclear cells from the liver of mice. As per the results, the proportion of CD3^+^CD4^+^T cells and CD3^+^CD8^+^T cells and M1 macrophages (F4/80^+^CD11c^+^) were found to be significantly increased in the liver of the HF-fed mice (Fig. 7A–C). In addition, we measured the expression of inflammatory cytokines, including TNF-α, IL-1β, and IL-6. The liver of the HF-fed mice showed a higher expression of TNF-α, IL-1β, and IL-6 (Fig. 7D). The plasma levels of TNF-α, IL-1β, and IL-6 were significantly higher in the HF-fed group than in the ND-fed group from 8 weeks (Figure S2C). These results suggest that a long-term HF diet can induce an inflammatory environment in the whole body, which may promote liver damage.

Malonaldehyde (MDA) is the end product of lipid peroxidation, reflecting the levels of oxidative stress in the liver. The content of MDA was significantly higher in the liver of the HF-fed group than in the ND-fed group (Fig. 7E). Next, we estimated the liver’s mRNA levels of fatty acid synthase. As shown in Fig. 7F, the expression of *FAS*, *ACC1*, *CD36*, *ChREBP*, and *SREBP1c* was significantly higher in the HF-fed mice. In addition, we have also analyzed the expression levels of lipid synthesis-related proteins in the liver. The results showed that the expression levels of FASN, ACC1, and CD36 in the HF-fed group were significantly higher than in the ND-fed group, and there was no significant difference in the expression levels of ChREBP and SREBP1 (Fig. 7G,H). Overall, these results suggest that HF can upregulate the expression of lipid synthesis-related proteins, thereby promoting liver inflammation and hepatic steatosis.

### FMT can alleviate the HF-induced metabolic disorders

**Figure 8:**
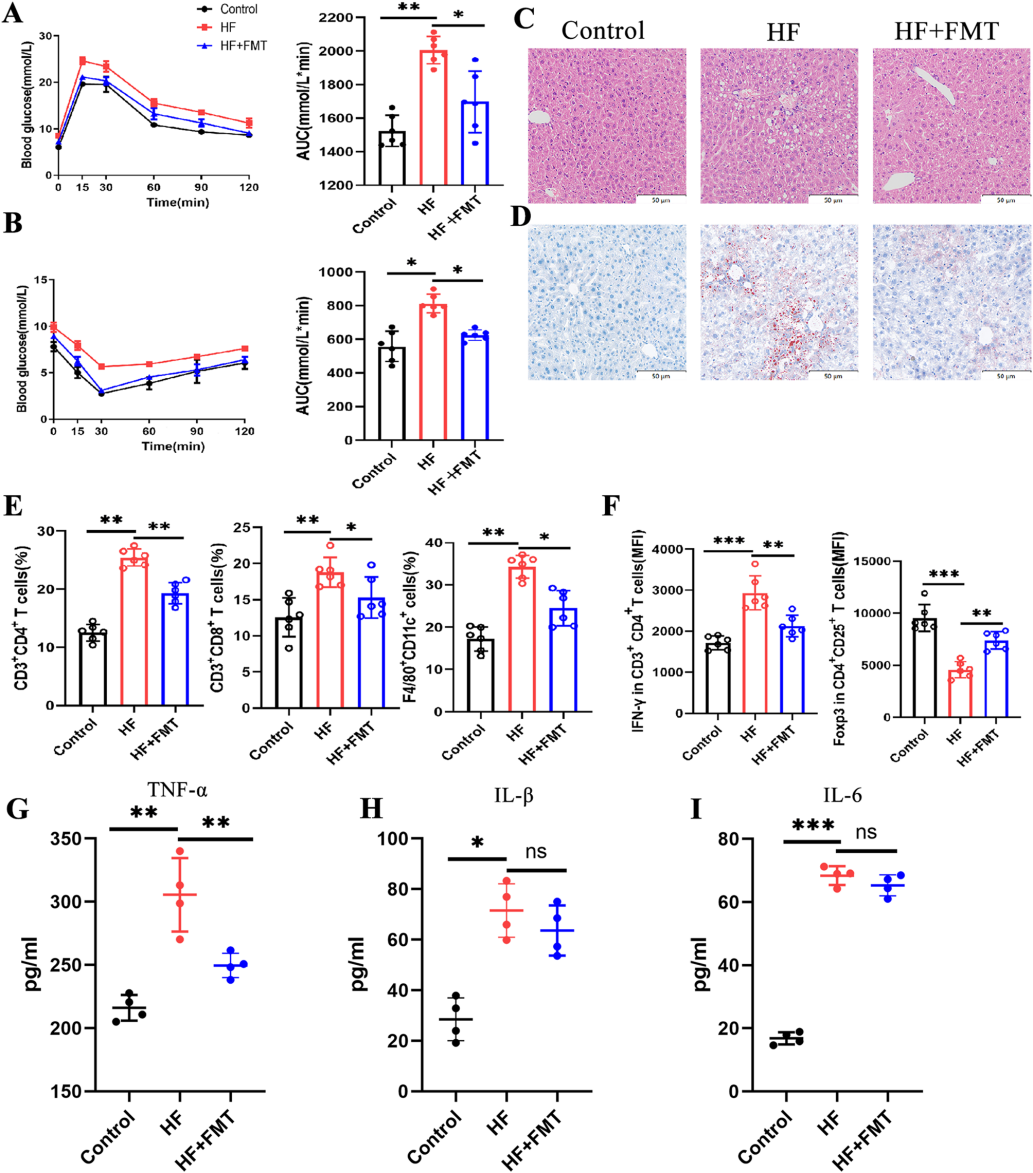
The effect of FMT on insulin resistance, liver histology, and immune homeostasis imbalance in HF-fed mice. (**A**) Glucose tolerance test (left) and glucose tolerance test calculated AUC (right) measured after FMT treatment. (**B**) Insulin tolerance test (left) and insulin tolerance test calculated AUC (right) measured after FMT treatment. Representative images of liver stained with (**C**) H&E and (**D**) Oil-red-O. Bar: 50 µm. (**E**) Flow cytometry analysis of CD3^+^CD4^+^ T cells, CD3^+^CD8^+^ T cells, and M1 macrophages (F4/80^+^CD11c^+^) in liver. (**F**) Flow cytometry analysis of the MFI expression level of Th1 cells (CD4^+^IFN-γ^+^) and Treg cells (CD25^+^Foxp3^+^) in MLN. The levels of TNF-α (**G**), IL-1β (**H**) and IL-6 (**I**) in serum were determined using ELISA. One-way analysis of variance (ANOVA) test was used for determining statistical significance. *p < 0.05; **p < 0.01.

To explore the therapeutic effects of FMT against metabolic disorder in the HF-fed mice, fecal samples or PBS were administered to mice for 4 weeks. Firstly, the OGTT and ITT were tested and the areas under the curve of glucose were calculated. It was observed that glucose intolerance and insulin resistance were improved in the HF + FMT group, again reverting to levels not significantly different from ND-fed mice (Fig. 8A,B). Plasma ALT, AST, TG, and TCHO levels were significantly lower in HF + FMT mice compared with mice fed the HF for 12 weeks (Table 1). Furthermore, FMT improved the histologic degree of severity as assessed by a decrease in hepatic steatosis and lipid accumulations compared with the HF-fed mice (Fig. 8C,D). To elucidate the immunological mechanism underlying FMT-induced improvement of systemic metabolic disorder. Tc, M1 macrophages, Th1, and Treg cells were quantified in liver and MLN. FMT decreased the proportion of hepatic CD3^+^CD4^+^T cells, CD3^+^CD8^+^T cells and M1 macrophages (F4/80^+^CD11c^+^) compared with HF-fed mice for 12 weeks (Fig. 8E). At the level of the MLN, HF + FMT mice showed decreased proportions of Th1 cells (CD4^+^IFN-γ^+^) compared with HF-fed mice, whereas the proportion of Treg cells (CD25^+^Foxp3^+^) increased (Fig. 8F). Moreover, plasma cytokine analysis showed an increase in TNF-α in HF + FMT mice, but failed to show any systemic changes in other T-cell–associated cytokines (Fig. 8G–I).

**Table 1:** Effects of fecal microbiota transplantation (FMT) on plasma biochemistry.

	Control	HF	HF + FMT
ALT (U/L)	52.21 ± 5.92	217.8 ± 12.49**	70.89 ± 10.09^**#**^
AST (U/L)	151.3 ± 18.40	528.3 ± 43.89*	246.6 ± 34.25^**#**^
TG (mmol/L)	0.62 ± 0.09	1.07 ± 0.03*	0.82 ± 0.03^**##**^
TCHO (mmol/L)	1.45 ± 0.12	3.65 ± 0.32*	1.96 ± 0.11^**#**^

Results are expressed as means ± SEM. *p < 0.05 vs. Control; **p < 0.01 vs. Control; ^**#**^*p* < 0.05 vs. HF; ^**##**^*p* < 0.01 vs. HF.

## Discussion

HF consumption is blameworthy for the development and progression of metabolic diseases, and overconsumption of fructose can lead to obesity, fat accumulation, and dyslipidemia. However, the underlying biological mechanisms remain unclear as most studies was only to examine one time point or investigate the additive effects of high-fructose and high-fat diets^15,16^. Hence, in this study, we investigated the effects of long-term consumption of HF on mice at different stages and found that damage to the colon and adipose tissues might be an early event and that liver damage may be a subsequent effect. Furthermore, our study demonstrates that a long-term HF diet alters the gut microbiota diversities, leading to T cells alterations in the liver and MLN, and resulting in hepatic steatosis.

Previous studies have demonstrated that the chronic HF diets can impair glucose tolerance and insulin tolerance and lead to NAFLD^17^. While the effect of HF on body weight remains controversial. Several studies found that HF diets increase body weight^18,19^. Do et al.^20^ suggested that a high-glucose or HF diet can cause metabolic disorders in mice without body weight change. Our study also found that chronic HF diet intake did not result in being overweight from the 1th week until the end of treatment but led to oral glucose intolerance and insulin resistance after 8 weeks. Excessive fructose intake can affect the liver^21^. As expected, fructose markedly increased the levels of plasma ALT and AST and hepatic TG and TCHO. It also led to severe hepatic steatosis and aggregation of lipid droplets after 12 weeks. However, the liver of mice in HF diet group did not have apparent vacuolar degeneration and lipid droplet aggregation at 4 weeks, suggesting that the short-term HF diet did not affect the liver. Interestingly, the white fat and brown fat cells were significantly enlarged, the Inflammation foci was appeared in white adipose tissue, the proportion of oversized adipocytes was significantly increased, the islet cells were disordered, and the length of the colon was significantly shortened in the HF diet group compared with the controls from the 4th week. Therefore, we indicated that colon, pancreas, and adipose tissue lesions may be an early event after an HF diet, and liver damage may be a subsequent effect. Next, we will further discuss the specific mechanism of the metabolic disorder caused by HF.

Phylogenetic and metagenomic analyses of gut microbiota have been extensively studied in the context of metabolic disorders. A growing body of evidence has demonstrated that obesity and its related diseases, such as dyslipidemia, inflammation and impaired glucose tolerance, are highly correlated with changes in gut microbiota^22,23^. Rong Tan et al.^16^ reported that an HF diet could increase *Firmicutes*/*Bacteroidetes* ratio. *Firmicutes* have been implicated in the development of diabetes and obesity^24,25^. In contrast, *Bacteroidetes* have normal effects on intestinal development, which produce molecules that mediate healthy immune responses and protect the host from inflammatory disease^26,27^. In this present study, we observed that an HF diet significantly reduced the richness and diversity of the gut microbiota. At the genus level, we observed more specific shifts, including the relative abundance of *Firmicutes*, such as *Blautia*, *Lachnoclostridium*, and *Oscillibacter* were significantly higher in the HF than in the ND-fed group. These data confirm and extent previous findings.

The gut microbiota regulates metabolism and plays an important role in the breakdown and absorption of nutrients. Numerous studies have shown a high correlation between the gut microbiota and gut barrier function^28,29^. As the intestine consumes a considerable amount of energy, modulating the intestinal volume and cellular architecture is deemed an important adaptation to fluctuations in nutrient availability^30,31^. Previous studies have suggested that an HF diet can alter the intestinal function. Samuel et al.^32^ reported that the length of intestinal villi was longer in HF-fed mice. Whereas our data showed that the length of small intestine villus, as well as the total length of the colon, decreased in the HF-fed group. These conflicting research findings may vary depending on the strain and age of the mice, the duration and the amount of HF feeding. Furthermore, it has been found that fructose metabolism in the gut leads to disruption of tight junction proteins^33^, which may account for the increased gut permeability and disruption of gut barrier function observed after fructose ingestion^34,35^. In this study, we demonstrated that the HF diet increased intestinal permeability and altered intestinal barrier characterized by decreased expression of tight junction proteins, such as ZO-1, occludin, and claudin-1. Impaired TJs can also lead to loose intercellular junctions, increased intestinal permeability, and even shedding of epithelial cells, leading to luminal macromolecular epithelial layer invasion and activation of inflammatory responses. The immune system is controlled by Treg cells, which express the transcription factor forkhead box protein P3 (Foxp3). Their primary function is to minimize excessive effector-T-cell activation and resultant tissue damage during infection-induced immunological responses^36,37^. Th1 cells are proinflammatory cells that are involved in adipose tissue inflammation associated with obesity-related pathologies^38^. We have found an increase in the proportion of pro-inflammatory Th1 cells and a decrease in the proportion of anti-inflammatory Treg cells in the intestinal lymph nodes of the HF-fed group. This imbalance in the ratio of Th1 and Treg cells may also be caused by changes in the gut microbiota. Additionally, compared with the control group, the jejunum, as the main part of fructose absorption, was found to have more CD4^+^ and CD8^+^ T cell infiltration in the HF-fed group. These results suggest that changes in the gut microbiota induced by an HF diet impair the intestinal mucosal barrier. Destruction of the integrity of the intestinal barrier increases the chances of various metabolites contacting the immune system, which may be the primary cause of hepatic steatosis caused by HF.

Importantly, NAFLD is no longer considered an exclusively hepatic disease, as multiple other organ systems participate in the pathogenesis^39^. Being on the most import one hand the gut, through dysregulation of the microbiome^40^. Increased intestinal permeability leads to the entry of gut-derived bacterial LPS into the portal vein, which is an essential trigger for fatty liver development^41^. Hepatic lipid accumulation upregulates hepatic pro-inflammatory cytokines by directly activating the TLR-4 pathway^42^. A recent report suggested that TNF-α plays a casual role in the onset of fructose-induced NAFLD/NASH and insulin resistance in mice^43^. IL-6 can induce B cell differentiation, antibody production, and T cell activation, proliferation, and differentiation^44^. IL-6 is known to be involved in the body’s immune response and initiates inflammation^45^. In our study, consistent with the histological and biochemical data, the levels of hepatic and plasmatic TNF-α, IL-6, and IL-1β were significantly elevated in fructose-exposed mice. Increased intestinal permeability can activate Kupffer cells through Toll-like receptor 4 (TLR-4) on the cell membrane, leading to liver inflammation^46^. We found that HF can significantly increase the proportion of CD3^+^CD4^+^ T cells, CD3^+^CD8^+^ T cells, and M1 macrophages in the liver. In addition, we found that the expression levels of FASN and ACC1, which are considered critical enzymes in adipogenesis, were significantly increased in the HF-fed group. CD36, ChREBP, and SREBP1c were increased in the HF-fed group compared to the ND-fed group. Fareeba et al.^47^ suggested that CD36 is essential in hepatic fat absorption and triglyceride storage. Therefore, an HF diet can induce higher expression of inflammatory cytokines and upregulate the protein expression levels of FASN, ACC1, CD36, ChREBP, and SREBP1c, thereby promoting the development of the fatty liver.

Next, to further investigate the mechanistic involvement of the observed alterations in T cells and macrophages in the pathogenesis of NAFLD in an HF diet mouse model, we sought to correct these disruptions using gut microbiota reconstitution. FMT effectively decreased CD3^+^CD4^+^ T cells, CD3^+^CD8^+^ T cells and M1 macrophages in liver and Th1 cells in MLN, whereas it increased Treg cells, thereby decreasing plasma ALT, AST, TG and TCHO, as well as histologically attenuating hepatic steatosis and lipid accumulations, thus improving NAFLD. The fact that the most pronounced effect on NAFLD was achieved by correcting an immune disruption at the level of the liver and MLN underlines the immense importance of gut microbiota in the pathogenesis of HF diet-induced hepatic steatosis and should encourage further exploration of the modulation of correcting gut microbiota in the treatment of metabolic disease.

The potential limitation of this study is that we are only simulate this phenomenon in animal models, so it is highly artificial. Further studies on clinical samples should be conceded. Hence, further studies on clinical samples are required to reveal the mechanism of this phenomenon.

In conclusion, our results suggested that an HF diet-induced hepatic steatosis may be caused by intestinal barrier damage and immune disorders mediated by an alteration in the gut microbiota. Intestinal structure impaired and intestinal inflammation may be an early event, and liver inflammation and hepatic steatosis may be a subsequent effect in HF-fed mice. Furthermore, FMT effectively improve systemic metabolic disorders by maintaining liver and intestinal immune homeostasis.

## Materials and methods

### Animals and diets

Animal experiments were performed according to the National Institutes of Health's standards for the care and use of experimental animals. Our study was approved by the Animal Welfare and Research Ethics Committee of Qingdao University (20220308C574020220907107). Methods are reported in accordance with ARRIVE guidelines.6 weeks-old male C57BL/6J mice were purchased from SPF (Beijing) BIOTECHNOLOGY Co., Ltd (SCXK-2017-0010). The animals were housed in animal rooms with a temperature of 23℃, humidity control (65%), 12 h light/12 h dark cycle, and food and water were provided ad libitum. Mice were given a one-week interval for domestication, accessing to food (standard food: 60% carbohydrate, 16% protein and 3% fat) and water freely. After this time, animals were randomly provided with two synthetic diets over a 12-week period. These were standard chow (60% carbohydrate, 16% protein, and 3% fat) and high fructose (60% fructose, 16% protein, and 3% fat). In the following, these two diets will be referred to as normal diet (ND) and high fructose (HF).

### Fecal microbiota transplantation (FMT)

The mice in the experimental groups were induced by an HF diet for 8 weeks and randomly divided into HF and HF + FMT groups, and the control group was fed a normal diet for 8 weeks. There were 10 mice in each group of HF group, HF + FMT group, and normal diet control group. During the experiment, the fresh feces of the healthy mice were collected within four hours in a sterile centrifuge tube, and immediately placed at − 80 °C and stored. Before transplantation, mice were treated for three consecutive days with 200 ul of an antibiotic cocktail (with each daily dose being administered by oral gavage after a 6 h fast) that contained 1 g/L ampicillin, 0.5 g/L neomycin, 0.5 g/L vancomycin, and 1 g/L metronidazole. The mice were given 100 μL of the microbiota suspension four times a week for four weeks. To prepare the microbiota suspension, 2–5 fresh feces pellets (80–100 mg) were resuspended with vortexing in 600 μL of reduced phosphate buffered saline (PBS). After resuspension, the tubes containing the feces in reduced PBS were centrifuged at 500×*g* for 1 min to remove insoluble material, and 100 μL of supernatant was administered to the mice by oral gavage. Mice in the HF group received the same antibiotics treatment and were transplanted only with reduced PBS^48^.

### Oral glucose tolerance tests and insulin tolerance tests

Oral glucose tolerance test (OGTT) was performed at 4, 8, and 12 weeks after fasting for 12 h. Oral glucose (1g/kg body weight) and blood glucose levels measured with a glucometer at 0, 30, 60, 90, and 120 min after glucose administration. For insulin tolerance test (ITT), mice were injected with insulin by intraperitoneal injection of 1U/kg body weight after fasting for 4 ~ 5 h, and their blood glucose was measured at 0, 30, 60, and 120 min after the injection.

### Analysis of fecal microflora composition by 16S rRNA gene sequencing

Fresh fecal samples of mice were collected at week 4 and immediately stored at − 80 °C until processing. For microbial community analysis, fecal DNA extraction and V3–V4 hypervariable region of the 16S rRNA gene amplification were carried out using a MiSeq according to the manufacturer’s instructions.

### Quantitative RT-PCR

RNA was extracted by RNEasy kit (Vazyme, Nanjing, China), the total RNA was transcribed in a volume of 1 μg by protocol (Vazyme, Nanjing, China). 2 × Unlversal SYBR Green Fast qPCR Mix (Vazyme) was used for qPCR and cDNAs were synthesized by using Superscript kit (Roche). The primers were synthesized by Sangon Biotech (Shanghai, China). The sequences of primers were listed in Table 2. The reaction procedure was as follows: initial denaturation at 95 °C for 30 s, followed by 40 cycles of 95 °C for 10 s (denaturation), 60 °C for 30 s (annealing and extension). The 2 − ΔΔCT method was used to determine the relative expression of the target genes normalized to GAPDH.

**Table 2:** Oligonucleotide sequences of primers.

Gene	Upstream (5'-3')	Downstream (5'-3')
FAS	TGCTTGCTGGCTCACAGTTAAGAG	TTTCACGAACCCGCCTCCTCAG
ACC1	TACCTTCTTCTACTGGCGGCTGAG	GCCTTCACTGTTCCTTCCACTTCC
CD36	GCAGGTCTATCTACGCTGTGTTCG	TGTCTGGATTCTGGAGGGGTGATG
ChREBP	TACGTCGGCAATGCTGACATGATC	TGGGAGGCGGGAGTTGGTAAAG
TNF-α	CGCTCTTCTGTCTACTGAACTTCGG	GTGGTTTGTGAGTGTGAGGGTCTG
IL-6	CTTCTTGGGACTGATGCTGGTGAC	TCTGTTGGGAGTGGTATCCTCTGTG
IL-1β	CACTACAGGCTCCGAGATGAACAAC	TGTCGTTGCTTGGTTCTCCTTGTAC
GAPDH	CCAGCAAGGACACTGAGCAA	GCCCCTCCTGTTATTATGGGG

### Western blotting

The liver and colon tissues were isolated and pestled, and were homogenized in radioimmunoprecipitation assay (RIPA) buffer. The mixture of 5 × loading buffer and lysate supernatant was 1:4 for Western blotting. The mixture was separated by SDS-PAGE and then transferred to the PVDF membrane. The membranes were blocked with 5% skim milk for 1 h at room temperature (25 ± 2 °C) and incubated with the primary antibodies overnight at 4 °C, and incubating peroxidase-labeled secondary antibodies 2 h at room temperature, and dropping electrochemiluminescence (ECL), and ECL, 1 min, signals were detected with chemiluminescence (Imagequant LAS500, Sweden). Primary antibodies: polyclonal rabbit anti-Occludin (1:1000, ABclonal, cat. no A12621), polyclonal rabbit anti-ZO-1 (1:1000, ABclonal, cat. no A11417), polyclonal rabbit anti-Claudin1 (1:1000, ABclonal, cat. no A11530), polyclonal rabbit anti-ACC1 (1:1000, ABclonal, cat. no A15606), monoclonal rabbit anti-CD36 (1:1000, ABclonal, cat. no A19016), polyclonal rabbit anti-FASN (1:1000, ABclonal, cat. no A0461), polyclonal rabbit anti-SREBP1 (1:1000, Abcam, cat. no ab28481), polyclonal rabbit anti-ChREBP (1:1000, ABclonal, cat. no A7630), HRP-conjugated monoclonal rabbit anti-mouse Tubulin (1:2000, ABclonal, cat. no AC008). Secondary antibodies used were horseradish peroxidase-conjugated sheep anti-rabbit (1:5000, ABclonal, cat. no AS014).

### Histological analysis

Histological analyses were performed after hematoxylin and eosin (H&E) staining. Liver, pancreas, small intestine, scapular brown adipose tissue (BAT) and abdomenl white adipose tissue (WAT) were fixed in 10% formalin after mice were sacrificed. 5 μm Paraffin embedded tissue sections were stained with Hematoxylin–Eosin and Frozen sections of formalin-fixed liver were stained with Oil-Red O using standard techniques. The changes of tissue were observed by a light microscope (from Olympus, Japan).

### ELISA

After the mice were anesthetized, the eyeballs were removed and about 0.5–0.8 mL of blood was collected. Put the blood into a centrifuge tube, let it stand for 2 h, and centrifuge (3000 r/min, 10 min), take the supernatant, transfer it into an EP tube. The serum levels TNF-α, IL-1β, and IL-6 were detected by ELISA kits according to the manufacturer’s instructions. Absorbance values were determined by microplate reader, and sample concentration was analyzed using the standard curves.

### Biochemical analysis

Mice were fasted for 12 h and euthanized after anesthesia. Pelltobarbitalum Natricum was used for anesthesia by intraperitoneal injection at a dose of 50 mg/kg. The levels of aspartate aminotransferase (AST), alanine aminotransferase (ALT), triglycerides (TG), and cholesterol (CHO) in blood serum were quantified by an automated analyzer (Chemray 240, Rayto, Shenzhen, China).

### Flow cytometry analysis

Mice were sacrificed, livers and mesenteric lymph nodes (MLN) were dissected out. The liver of the mouse was ground and passed through 200 mesh copper mesh. Cell suspension was collected and centrifuged for 1 min at 650 rpm/min. After centrifugation, transfer the supernatant to a new centrifuge tube, add 5 mL of erythrocyte lysate, incubate on ice for 10 min, 1300 rpm/min, centrifuge for 5 min. Discard the supernatant, add 8 mL of freshly prepared 40% Percoll separation solution to the cell precipitation, resuspend the cells, 2500 rpm/min, centrifuge for 25 min, and the resulting cell precipitation is liver mononuclear cells. Collected liver mononuclear cells were surface-stained with PB450 anti-mouse CD3 (BioLegend, cat. 100214) and BV605 anti-mouse CD4 (BioLegend, cat. 100451) or PB450 anti-mouse CD3 and APC anti-mouse CD8a (BioLegend, cat. 100714) or PE-Cy7 anti-mouse F4/80 (BioLegend, cat. 12311) and FITC anti-mouse CD11c (BioLegend, cat. 117306) for 30 min at 4 °C.

Collected intestinal lymph node lymphocytes were resuspended in RPMI 1640 medium containing 10% FBS.1 × 10^6^ cells per well were plated in 96-well plates, and stimulated with PMA and Ionomycin (MULTI SCIENCE, CS1001) at a concentration of 0.05ug/mL for 5 h. Then, BFA (Biolegend, cat. 420601) was added to block the secretion of intracellular cytokines for 4 h. Cells were washed with PBS to remove blocking agent and were surface-stained with PB450 anti- mouse CD3 and BV605 anti-mouse CD4 or BV605 anti-mouse CD4 and APC anti-mouse CD25 (BioLegend, cat. 102012) for 30 min at 4 °C. These cells were fixed in 4% paraformaldehyde for 8 min at room temperature. After washing, the cells were suspended with 0.2% Triton X and incubated for 30 min at 4 °C. The surface- stained CD3 and CD4^+^ T cells were then stained with PE anti-mouse IFN-γ. The surface-stained CD4 and CD25^+^ T cells were then stained with PE anti-mouse Foxp3 (BioLegend, cat. 320008)^49^. The fluorescent-labelled anti-mouse monoclonal antibodies were processed following the manufacturer's instructions. Finally, the cells were analysed using Beckman CytoFLEX, and the data were analysed using FlowJo v10.

### Liver MDA activity assay

Take 10% of mouse liver tissue, mechanically homogenize it in an ice-water bath to prepare a 10% homogenate. 2500–3000 rpm, centrifuged for 10 min, and the supernatant was taken for determination by enzyme-linked immunosorbent assay.

### Immunohistochemistry staining

The jejunum and brown adipose tissues were fixed with 4% formalin and embedded in paraffin. Paraffinem-bedded sections (5 μm) deparaffinized, rehydrated, and steaming water 5 min, antigen retrieval with sodium citrate buffer (pH 6.0) for 6 min with pressure cooker. Endogenous catalase was inactivated with 3% hydrogen peroxide. Then, sections were incubated with anti-mouse CD4 (1:200, Novus, cat. NBP1-19371), anti-mouse CD8 (1:100, Novus, cat. NB200-578), anti-mouse UCP1 (1:100, ABclonal, cat. no A5857), and anti-mouse PGC-1α (1: 200, BOSTER, cat. no BA2816-1), overnight at 4 °C. Following morning, sections were incubated with the secondary antibodies in the incubator 40 min. After washed with distilled water and PBS, then sections were exposed to DAB, 1 min; and hematoxylin, 1 min; differentiate solution, 1 s; and observed after the neutral gum seal^50^.

### Statistical analysis

Graphpad prism 8 was used to analyze the data, and statistical significance was determined by a unpaired Student’s t-test or Mann–Whitney test. One-way analysis of variance (ANOVA) test was used for multiple comparisons. The significance level was set at *p* < 0.05. Statistical significance was also taken as *p < 0.05; **p < 0.01; ***p < 0.001.

## Supplementary Information

Supplementary Information.

## Data Availability

All data used to support the findings of this study are available from the corresponding author upon request.
