# Supplementary material for: Gut microbiota induces hepatic steatosis by modulating the T cells balance in high fructose diet mice
Source: Sci Rep. 2023 Apr 24;13:6701. doi: 10.1038/s41598-023-33806-8 (PMC10126116; doi:10.1038/s41598-023-33806-8)
Supplement: Supplementary file 1 — Supplementary Information. [file 41598_2023_33806_MOESM1_ESM.docx]

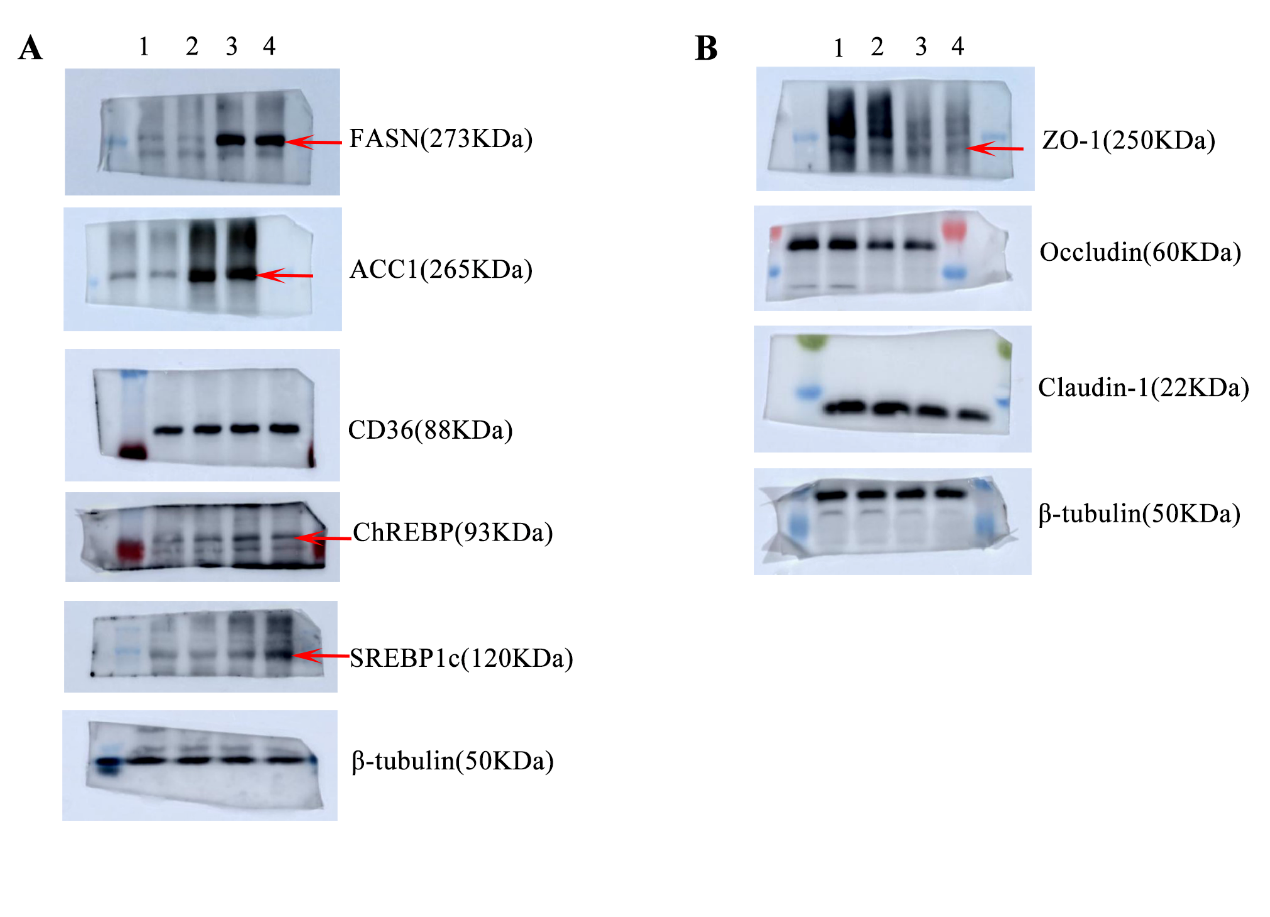


**Fig S1. (A)** Representative images of Western blots for lipid metabolism (FASN, ACC1, CD36, ChREBP, and SREBP1c) in liver. **(B)** Representative images of Western blots for tight junction proteins (ZO-1, occludin, and claudin-1) in colon.The results of WB showed that the channels 1–2 were the ND-fed group and the channels 3–4 were the HF-fed group. They are cut out from different parts of the same gel.


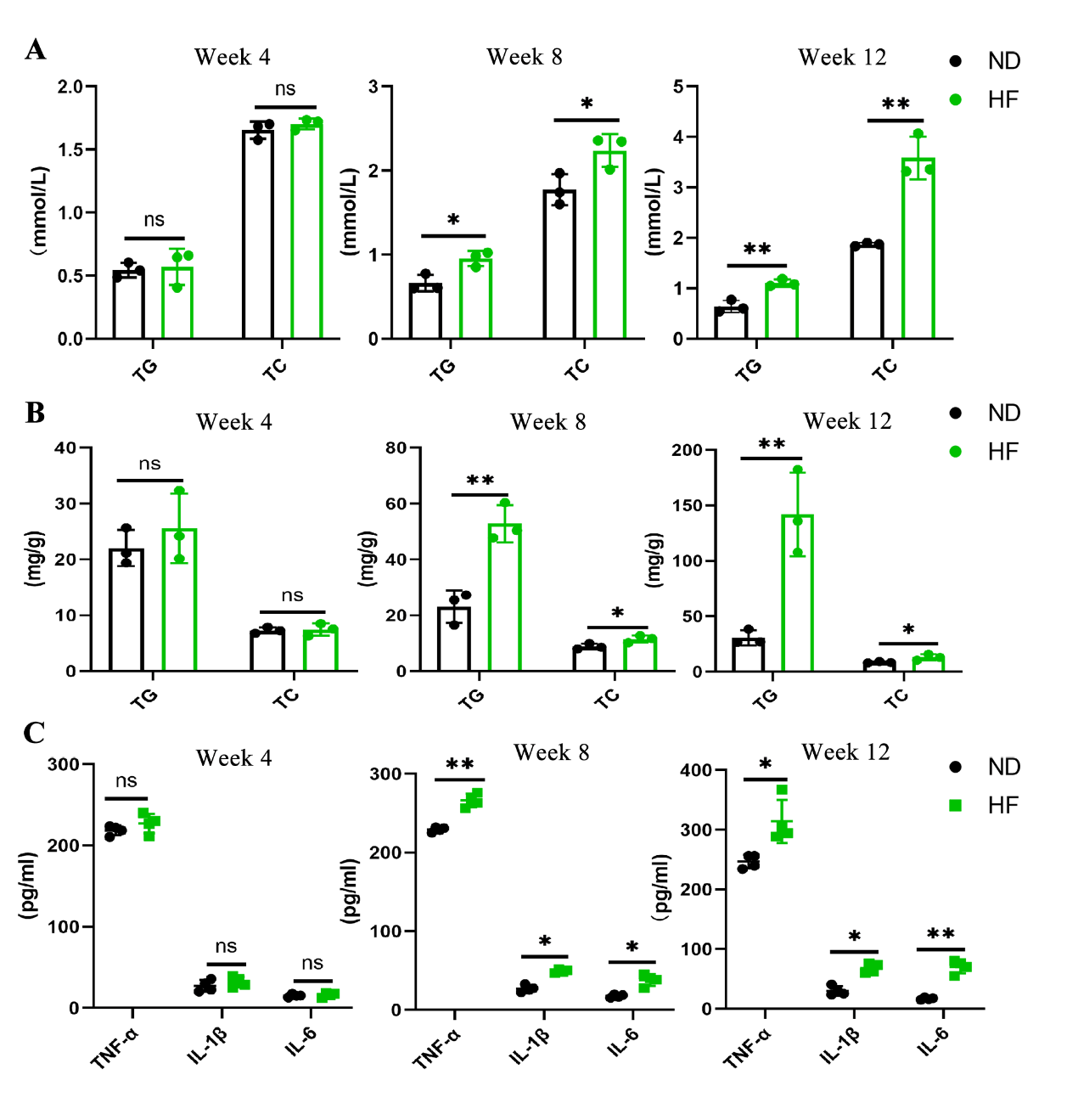


**Fig S2. (A)** Evolution of triglycerides (TG) and total cholesterol (TC) in serum during the study. **(B)** Evolution of triglycerides (TG) and total cholesterol (TC) in the liver. **(C)**The levels of TNF-α, IL-1β, and IL-6 in serum were determined using ELISA at 4,8, and 12 weeks. The student’s t-test was used to determine statistical significance. *, *p* < 0.05; **, *p* < 0.01.


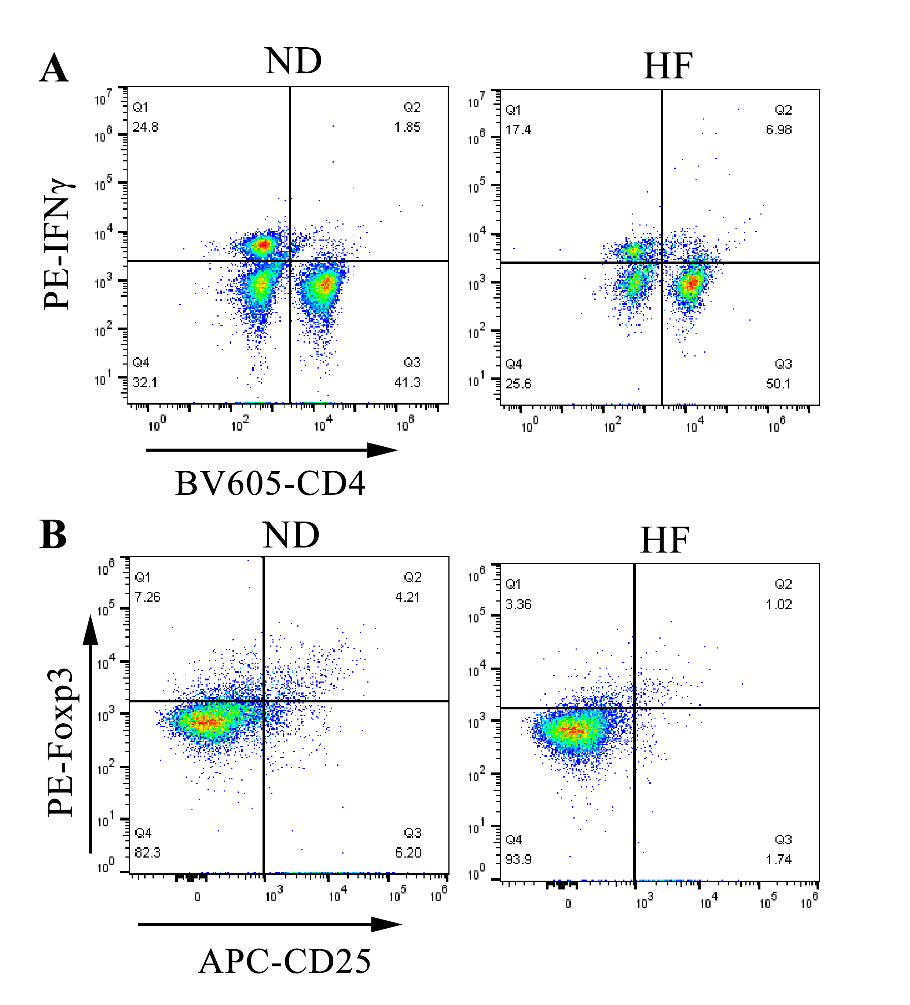


**Fig S3.** We gated on mesenteric lymph nodes lymphocytes at 4 weeks and measured **(A)** Th1 cells (CD4^+^IFN-γ^+^) and **(B)** Treg cells (CD25^+^Foxp3^+^) by flow cytometry.
